# Development and Application of Two Rapid Molecular Detection Assays for *Hyblaea puera* Cramer (Lepidoptera: Hyblaeoidea), a Major Pest of Mangroves and Teak

**DOI:** 10.3390/biology15060473

**Published:** 2026-03-15

**Authors:** Shengbo Zhao, Dezhi Kong, Yunpeng Liu, Qinghua Wang, Yaojun Zhu, Liangjian Qu

**Affiliations:** 1 Key Laboratory of Forest Protection of National Forestry and Grassland Administration, Ecology and Nature Conservation Institute, Chinese Academy of Forestry, Beijing 100091, China; nzfys06@163.com (S.Z.); kongdz@caf.ac.cn (D.K.); 15993311970@163.com (Y.L.); wqh633@caf.ac.cn (Q.W.); 2 Co-Innovation Center for Sustainable Forestry in Southern China, Nanjing Forestry University, Nanjing 210095, China; 3 Zhanjiang National Research Station for Mangrove Wetland Ecosystem, Zhanjiang 524448, China; yaojunzhu@gmail.com

**Keywords:** *Hyblaea puera*, molecular detection, species-specific PCR, LAMP

## Abstract

The teak defoliator, *Hyblaea puera*, native to South Asia and Southeast Asia (e.g., India, Laos, and Myanmar), has recently caused frequent outbreaks in mangrove forests across Guangdong, Guangxi, and other regions of China. Its larvae feed extensively on the leaves of *Avicennia marina*, severely threatening local mangrove ecosystems. However, accurate morphological identification of *H. puera* across its eggs, larvae, and pupae remains challenging. Therefore, the development of rapid molecular detection methods is essential for effective pest identification and monitoring, thereby supporting timely management interventions. In this study, mitochondrial protein-coding genes (PCGs) were analyzed from *H. puera* and related species were analyzed. Sliding window analysis was conducted to estimate nucleotide diversity (*Pi*), leading to the selection of the cytochrome c oxidase subunit I (*COI*) gene as the optimal target. Species-specific primers were designed based on the *H. puera COI* sequence, and two molecular detection assays—SS-PCR and LAMP—were developed. Both assays exhibited high specificity, stability, and sensitivity, successfully amplifying target fragments from *H. puera* across all tested geographic populations and different developmental stages. The limit of detection of the SS-PCR method was 83 fg/µL DNA, while that of the LAMP method reached 8.3 fg/µL DNA. The newly developed assays offer reliable and robust tools: the SS-PCR method is suitable for precise, large-scale detection in laboratory settings, whereas the LAMP assay is preferable for rapid, field-based detection of *H. puera*. These methods contribute to the early detection and effective management of *H. puera* populations, thereby safeguarding mangrove ecosystems.

## Simple Summary

The frequent outbreaks of *Hyblaea puera* represent a growing threat to mangrove ecosystems in China. Accurate identification of its immature stages (eggs, larvae, and pupae), however, remains challenging due to the inherent constraints of conventional morphological approaches. To address this, we aimed to develop a rapid and reliable molecular detection technique to facilitate timely and effective pest monitoring and management. After evaluating mitochondrial protein-coding genes, the mitochondrial cytochrome c oxidase I (*COI*) gene was selected as an optimal molecular marker. Based on this target marker, we established two specific detection assays: species-specific PCR (SS-PCR) and loop-mediated isothermal amplification (LAMP). Both methods exhibited high specificity and successfully distinguished *H. puera* from sympatric non-target species. These novel molecular tools enable forest managers to allocate pest control resources and equipment in a targeted manner, avoiding the waste of blanket treatments and improving the cost-effectiveness of the prevention and control of *H. puera*, thereby supporting the protection of economically and ecologically important forests and mangrove ecosystems from this invasive pest.

## 1. Introduction

Forest biological invasions, as a widespread global phenomenon, pose unprecedented threats to biodiversity and forestry resources [1,2]. In China, major invasive species have caused severe damage to forest ecosystems, adversely affecting species diversity and ecological stability, with impacts often exceeding those of native pests [3,4]. Consequently, research on potential invasive species has become a critical priority in contemporary forestry science. Timely detection and accurate identification are fundamental prerequisites for developing effective management strategies and quarantine measures [5].

*Hyblaea puera* (Lepidoptera: Hyblaeidae), commonly known as the teak defoliator, is a pest native to South and Southeast Asia, including India, Laos, Thailand and Myanmar. It primarily infests teak (*Tectona grandis*) and the grey mangrove (*Avicennia marina*) [6,7,8]. The species was first recorded in China in 1975, initially confined to major teak cultivation zones [9]. In 2010, *H. puera* was observed infesting mangrove forests in Guangxi, feeding on *A. marina* leaves. A large-scale outbreak occurred in 2015, affecting approximately 300 hectares of mangrove stands and resulting in extensive defoliation and mortality of *A. marina*. With a short developmental period, *H. puera* can complete up to 11 generations per year in Guangxi’s coastal areas, posing a serious and ongoing threat to mangrove ecosystems in China [10,11]. The pest has since spread to multiple provinces, including Shaanxi and Gansu in northern China, demonstrating a distinct northward expansion from its original southern range [12].

Accurate identification of *H. puera* across its egg, larval, and pupal stages remains challenging. Although combining morphological methods with DNA barcoding is a viable approach, it requires specialized taxonomic expertise and laboratory equipment, is time-consuming, and is limited by the coverage and accuracy of existing reference databases [13]. Furthermore, specimens collected from light traps in mangrove areas are often decomposed or degraded, significantly compromising reliable identification. In contrast, species-specific PCR (SS-PCR) targeting single genes is an efficient and reliable molecular detection method that has been widely adopted for identifying important invasive species [14,15]. Mitochondrial genes are preferred as targets for SS-PCR development due to their maternal inheritance, high copy number, conserved structure, and lack of recombination [16,17]. However, despite the simplicity and efficiency of SS-PCR, its laboratory infrastructure limits its application, particularly for the on-site identification of *H. puera*.

Loop-mediated isothermal amplification (LAMP), first developed by Notomi et al., is a widely used nucleic acid amplification technique [18]. Compared with conventional amplification methods, LAMP offers greater operational simplicity, higher specificity and sensitivity, and simplified result visualization. Although initially prominent in medical diagnostics [19], LAMP has demonstrated promising potential in entomology in recent years [20]. However, its high sensitivity also makes it prone to aerosol contamination, and the required visualization reagents may pose safety concerns and increase operational costs [21]. Consequently, the parallel development of both SS PCR and LAMP methods would better accommodate detection and monitoring across varied scenarios.

Currently, the lack of a rapid field detection method for *H. puera* poses a significant challenge to effective monitoring systems. Therefore, there is an urgent need to develop cost-effective, equipment-free, and visual detection assays to support frontline quarantine and pest management. To meet the practical needs of different testing scenarios, this study aims to establish and optimize two complementary detection protocols, facilitating a stratified screening strategy tailored to laboratory and field settings.

Based on the phylogenetic tree established by Shah et al. [22] using mitochondrial genomes of *H. puera* and related species, this study selected closely related species for comparative analysis. Mitochondrial protein coding gene (PCG) sequences were extracted, and sliding window analysis was performed to calculate nucleotide diversity (Pi) values [23]. This approach enabled the selection of an optimal target gene for developing both SS-PCR and LAMP assays, supporting more rapid and accurate identification of *H. puera*.

## 2. Materials and Methods

### 2.1. Sample Collection and DNA Extraction

Larval specimens of *H. puera* were collected from four sites across Guangxi, Guangdong, and Yunnan provinces between June and August 2024 (Table 1). The larvae were reared on their respective host plants until adult emergence. Based on morphological characteristics, the adults were identified as *H. puera* [10]. The adults were then preserved in absolute ethanol and stored at −80 °C. Additionally, egg, larval, and pupal samples of *H. puera* were provided by the Insect Pathology and Entomopathogen Research Group, Ecology and Nature Conservation Institute, Chinese Academy of Forestry. The following species were included as experimental controls: *Spodoptera frugiperda*, *Spodoptera litura*, *Mythimna separata*, *Ostrinia furnacalis*, *Cnaphalocrocis medinalis*, *Plodia interpunctella*, and *Dichocrocis punctiferalis*. Genomic DNA was extracted from all samples using the DNA extraction kit (Tiangen Biotech (Beijing) Co., Ltd., Beijing, China, No. A0116A) following the manufacturer’s instructions.

### 2.2. Selection of Target Gene

Based on the phylogenetic tree reconstructed by Shah et al. [22], the mitochondrial genomes of *Hyblaea puera* (GenBank: MW885970) and four related species from the family Crambidae—*Chilo suppressalis* (GenBank: MK207057), *Diatraea saccharalis* (GenBank: FJ240227), *Ostrinia furnacalis* (GenBank: MN793323), and *Ostrinia nubilalis* (GenBank: AF442957)—were retrieved from the NCBI database. The 13 PCGs were extracted and aligned using Geneious Prime 2025 software. Sliding window analysis was conducted using DnaSP v6 software, employing a window size of 200 bp and a step size of 20 bp [21]. The gene exhibiting the lowest nucleotide diversity (*Pi*) value was selected as the target marker.

### 2.3. Design of H. puera-Specific Primers and PCR Protocol

The *COI* gene sequences of the selected species described in Section 2.2 were extracted and aligned using Geneious Prime 2025 software. Regions exhibiting high intra-species conservation within *H. puera* and high inter-species specificity against non-target species were identified. (Figure S1) Based on these regions, three pairs of specific primers were designed (Table 2). All primers were synthesized by Tsingke Biotechnology Co., Ltd. (Beijing, China).

PCR amplification was carried out in a 25 µL reaction mixture containing 2 µL of DNA template (832 ng/µL), 1 µL each of forward and reverse primers (10 µM), 12 µL of DNA Polymerase (Takara Bio Inc., Kusatsu, Japan No. RR370A), and 9 µL of ddH_2_O. The temperature-gradient PCR cycling conditions were as follows: initial denaturation at 95 °C for 5 min, followed by 20, 25 or 30 cycles of denaturation at 95 °C for 30 s, and annealing for 30 s across a gradient from 55 °C to 58 °C (with individual reactions set at 1 °C intervals), and extension at 72 °C for 30 s; with a final extension at 72 °C for 10 min. A negative control using ddH_2_O instead of DNA template was included in each run.

To visualize the amplification products, a 9 µL aliquot of each PCR product was mixed with 1 µL of 10× loading buffer and separated on a 1% agarose gel submerged in 1× TAE buffer. Electrophoresis was conducted at 80 V for 10–15 min. The gel was subsequently visualized and photographed using a gel imaging system.

### 2.4. Validation of Specificity and Sensitivity of the SS-PCR Assay

The quality of extracted genomic DNA from all insect samples was verified by amplification using the universal *COI* gene primers LCO1490 (5′-GGTCAACAAATCATAAAGATATTGG-3′) and HCO2198 (5′-TAAACTTCAGGGTGACCAAAAAATCA-3′). All samples yielded an amplification product of approximately 650 bp, confirming the integrity of the DNA templates for subsequent PCR analyses. The PCR reagents and cycling conditions were identical to those described in Section 2.3, with the exception that the annealing temperature was adjusted to 43 °C.

The specificity of the SS-PCR assay was primarily assessed by testing DNA templates from *H. puera* against those from non-target control species. The robustness of the assay was further verified using DNA from *H. puera* samples extracted from distinct geographical populations and at different developmental stages. To determine the sensitivity of the assay, the DNA of *H. puera* was subjected to 10-fold serial dilutions. Each experiment was conducted with three independent replicates. The concentrations in the dilution series were: 83.2 ng/µL, 8.32 ng/µL, 0.83 ng/µL, 83 pg/µL, 8.3 pg/µL, 0.83 pg/µL, 83 fg/µL, 8.3 fg/µL, and 0.83 fg/µL.

### 2.5. Design of H. puera-Specific LAMP Primers and Reaction Setup

LAMP primers were designed using the web-based Primer Explorer V5 tool (available at http://primerexplorer.jp/ (accessed on 10 July 2025)). The target gene sequence was input in FASTA format, and the primer design parameters were adjusted to meet specific experimental requirements. The primers F3 and B3 exhibited a Tm range of 54–56 °C, with 3′-end stability (ΔG) values ≤ −4 kcal/mol and a GC content of 40–50%. Based on these results, an optimal primer set was selected, comprising two pairs of primers: outer primers (F3 and B3) and inner primers (FIP and BIP) (Table 3). All primers were synthesized by Tsingke Biotechnology Co., Ltd. (Beijing, China).

To prevent aerosol contamination, 50 µL of liquid paraffin was overlaid on the reaction mixture in each tube. The LAMP reaction was performed in a total volume of 25 µL, consisting of 12 µL of 2× BcaBest Buffer, 2 µL of 10× LAMP Primer Mix, 1 µL of BcaBest DNA Polymerase, 2 µL of DNA template (Takara Bio Inc., RR380A), and 8 µL of ddH_2_O.

The temperature-gradient LAMP reaction was performed across a gradient from 60 °C to 65 °C (with individual reactions set at 1 °C intervals) for 30 min, followed by enzyme inactivation at 85 °C for 15 min. Subsequently, 1 µL of a 1:10 dilution of 10,000× SYBR Green I dye (Beijing Solarbio Science & Technology Co., Ltd., Beijing, China, SY1020) was added to each tube. Results were visualized based on the color change in the mixture: green indicated a positive result, while orange denoted a negative result.

### 2.6. Specificity and Sensitivity Validation of the LAMP Assay

The specificity and sensitivity of the LAMP assay were validated following the same procedures described in Section 2.4.

### 2.7. Establishment of the LAMP-LFD Assay for H. puera

To enable detection via lateral flow dipstick (LFD), the primers were modified by labeling the 5′ end of the FIP primer with Biotin and the 5′ end of the BIP primer with FITC. LFD test strips were obtained from Amprobe Future (Changzhou) Biotechnology Co., Ltd., Changzhou, China (No. WLFS8204).

Following LAMP amplification, 10 µL of the reaction product was diluted with 190 µL of sterile water and vortexed thoroughly. Subsequently, 80 µL of the diluted sample was applied to the sample port of the LFD strip. Results were visualized approximately 1–2 min after the control line (C line) became visible. Results were considered valid only if observed within 15 min of the control line appearing.

## 3. Results

### 3.1. Selection of the Target Gene Based on Nucleotide Diversity

**Figure 1:**
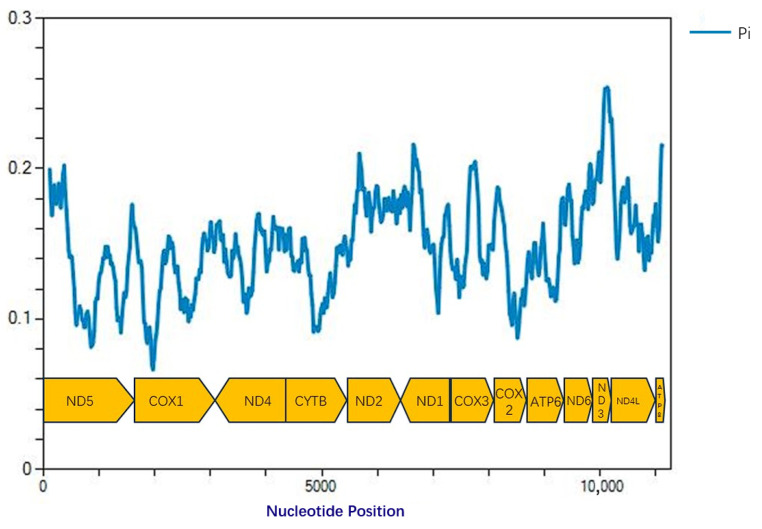
Nucleotide diversity (*Pi*) values of the 13 protein-coding genes (PCGs).

The nucleotide diversity (*Pi*) values of the 13 protein-coding genes (PCGs) across the 5 mitochondrial genomes were analyzed. The *Pi* values for individual genes ranged from 0.12 to 0.20. The three genes showing the highest Pi values were *ND2* (0.18), *ND6* (0.20), and *ATP8* (0.20), whereas those with the lowest values were *COI* (0.12), *COII* (0.13), and *ND5* (0.14) (Figure 1). Given that the *COI* gene exhibited the lowest nucleotide diversity, it was selected as the target gene for developing the rapid detection assays.

### 3.2. Specificity, Stability, and Sensitivity of the SS-PCR Assays

**Figure 2:**
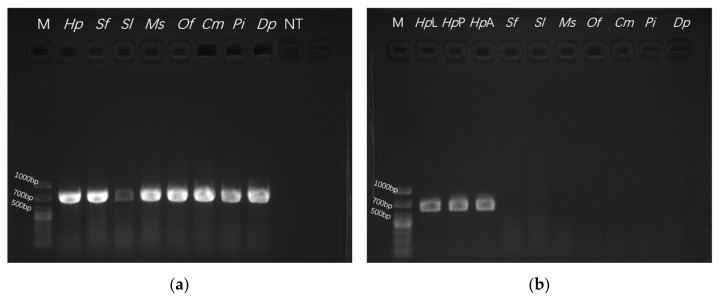

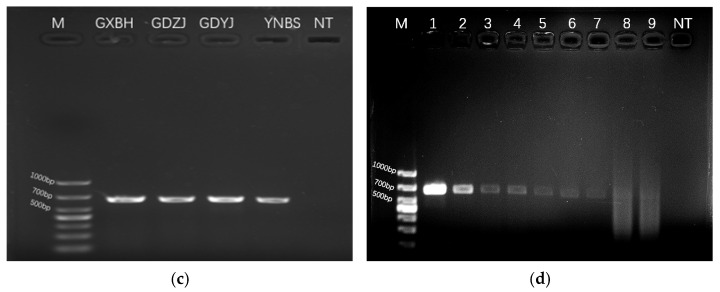
(**a**) PCR amplification products using universal *COI* primers; (**b**) Specificity test of the HPCOI-3 primer set; (**c**) Stability test of the HPCOI-3 primer set; (**d**) Sensitivity test of the HPCOI-3 primer set. M: D1000 Marker; *Hp*: *H. puera*; *Sf*: *S. frugiperda*; *Sl*: *S. litura*; *Ms*: *M. separata*; *Of*: *O. furnacalis*; *Cm*: *C. medinalis*; *Pi*: *P. interpunctella*; *Dp*: *D. punctiferalis*; NT: no template control; *Hp*L: *H. puera* larvae; *Hp*P: *H. puera* pupae; *Hp*A: *H. puera* adult; 1: 83.2 ng/µL; 2: 8.32 ng/µL; 3: 0.83 ng/µL; 4: 83 pg/µL; 5: 8.3 pg/µL; 6: 0.83 pg/µL; 7: 83 fg/µL; 8: 8.3 fg/µL; 9: 0.83 fg/µL.

Genomic DNA from *H. puera* and related species was successfully amplified using universal *COI* primers, yielding a product of approximately 650 bp (Figure 2a). This confirmed the integrity and suitability of the DNA templates for subsequent experiments. Three primer pairs targeting the *COI* gene were designed and evaluated.

The optimal annealing temperature for PCR was established at 58 °C, as lower temperatures significantly increased non-specific amplification. The cycle number was optimized to 25, since fewer cycles compromised detection sensitivity, while more cycles promoted primer-dimer formation. In specificity assays, Primer Set 3 (HPCOIF-3/HPCOIR-3) demonstrated high specificity, amplifying a distinct 602 bp fragment only from *H. puera*. No amplification was observed for the non-target species: *S. frugiperda*, *S. litura*, *M. separata*, *O. furnacalis*, *C. medinalis*, *P. interpunctella*, and *D. punctiferalis* (Figure 2b). The stability of the assay was assessed using DNA extracted from diverse geographical populations and various developmental stages of *H. puera*. Primer Set 3 consistently produced clear amplification across all samples (Figure 2c). Sensitivity assays, performed using 10-fold serial dilutions of *H. puera* DNA, established the detection limit of Primer Set 3 at 83 fg/µL, indicating sufficient sensitivity for rapid detection requirements (Figure 2d). Original agarose gel electrophoresis images for all replicates are available in Figure S2.

### 3.3. Specificity, Stability, and Sensitivity of the LAMP Assay and Optimization of Reaction Conditions

**Figure 3:**
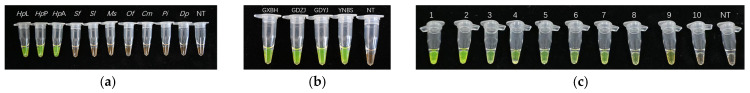
(**a**) Results of the LAMP specificity test; (**b**) Results of the LAMP stability test; (**c**) Results of the LAMP sensitivity test. *Hp*: *H. puera*; *Sf*: *S. frugiperda*; *Sl*: *S. litura*; *Ms*: *M. separata*; *Of*: *O. furnacalis*; *Cm*: *C. medinalis*; *Pi*: *P. interpunctella*; *Dp*: *D. punctiferalis*; NT: no template control; 1: 83.2 ng/µL; 2: 8.32 ng/µL; 3: 0.83 ng/µL; 4: 83 pg/µL; 5: 8.3 pg/µL; 6: 0.83 pg/µL; 7: 83 fg/µL; 8: 8.3 fg/µL; 9: 0.83 fg/µL; 10: 83 ag/µL.

The reaction temperature was experimentally screened and determined to be optimal at 65 °C, as lower temperatures led to insufficient color development, thereby hindering accurate result interpretation. Specificity assays demonstrated that reactions containing *H. puera* DNA templates produced positive results (visualized as a color change) upon indicator addition. In contrast, reactions with DNA from all non-target species (*S. frugiperda*, *S. litura*, *M. separata*, *O. furnacalis*, *C. medinalis*, *P. interpunctella*, *D. punctiferalis*) remained negative, confirming the high specificity of the assay (Figure 3a). Stability assays using DNA from different geographical populations and various developmental stages of *H. puera* demonstrated that the LAMP primers consistently produced clear positive results (Figure 3b). Sensitivity evaluations established the detection limit of the LAMP assay at 8.3 fg/µL, reflecting high sensitivity sufficient for rapid detection (Figure 3c).

**Figure 4:**
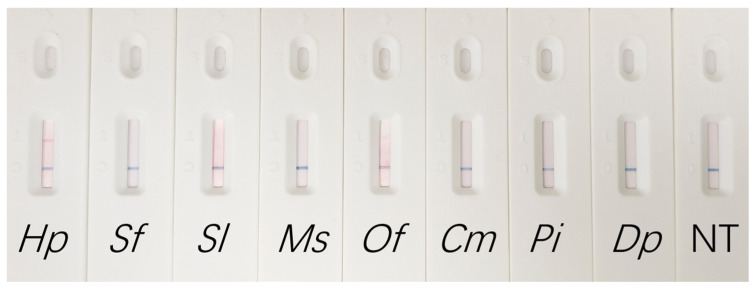
LAMP-LFD detection results. *Hp*: *H. puera*; *Sf*: *S. frugiperda*; *Sl*: *S. litura*; *Ms*: *M. separata*; *Of*: *O. furnacalis*; *Cm*: *C. medinalis*; *Pi*: *P. interpunctella*; *Dp*: *D. punctiferalis*; NT: no template control.

Furthermore, using primers labeled with Biotin (FIP) and FITC (BIP), amplification products were successfully visualized on a lateral flow dipstick. The LAMP-LFD assay yielded positive results exclusively for *H. puera* DNA, while all non-target species tested negative. This confirmed that the specificity of the assay was maintained on the LFD platform. These results demonstrate the effective integration of LFD with LAMP, establishing a rapid and cost-effective detection platform (Figure 4).

## 4. Discussion

Effective management of *H. puera* relies heavily on the ability to detect infestations before population outbreaks, particularly given the pest’s migratory behavior and rapid population growth. Our field surveys across Guangdong, Guangxi, and Yunnan provinces revealed abrupt population fluctuations and seasonal variations, consistent with the migratory patterns previously documented in India [24,25,26,27]. To address the critical need for early warning systems, this study developed and evaluated two distinct molecular diagnostic tools: a species-specific PCR (SS-PCR) assay and a loop-mediated isothermal amplification (LAMP) assay coupled with lateral flow dipstick (LFD) visualization. While both assays successfully distinguished *H. puera* from seven sympatric lepidopteran species, a critical comparison reveals distinct performance profiles and suitability for different scenarios.

The SS-PCR assay demonstrated exceptional specificity and stability, consistently amplifying a distinct 602 bp fragment of the *COI* gene across all tested developmental stages and geographical populations of the target species. Its key advantage lies in the robustness of conventional thermal cycling, a technique regarded as the gold standard for accuracy under controlled laboratory conditions. This method involves standard procedures such as PCR amplification and DNA fragment analysis, making it well suited for diagnostic applications in fully equipped laboratories. However, it is not appropriate for rapid, on-site testing in field settings. Furthermore, the detection limit of SS-PCR was determined to be 83 fg/µL, indicating considerably lower sensitivity compared to the LAMP method.

The LAMP assay for *H. puera* exhibited significantly higher sensitivity, with a detection limit of 8.3 fg/µL representing a tenfold improvement over the SS-PCR method. Such high sensitivity is comparable to, or even exceeds, that of isothermal amplification methods developed for other invasive pest detection (e.g., RPA for *Hylurgus ligniperda* or LAMP for *Spodoptera frugiperda*), which typically operate within the femtogram (fg) range [28,29]. The capacity to detect DNA at such low concentrations is critical for early identification of infestations, where target material is often limited. Furthermore, conventional colorimetric LAMP assays may be influenced by subjective visual interpretation, whereas the integration of LFD visualization provides more definitive results by determining the presence or absence of the test line. The LAMP-LFD assay eliminates the need for thermocyclers and gel electrophoresis, effectively overcoming the operational constraints of field-based diagnostics and providing a rapid, cost-effective, and efficient detection method for on-site testing.

With its femtogram-level sensitivity, the LAMP-LFD assay enables the detection of trace samples, including a single egg, early-instar larvae, or even environmental DNA (eDNA) residues potentially deposited on host plants. This high sensitivity is especially critical for the routine surveillance of *H. puera*, a highly mobile pest whose strong flight capacity demands rapid and sensitive detection methods. Moreover, the assay consistently performs well across individuals from diverse geographic origins and all life stages, ensuring reliable detection regardless of the pest’s source or developmental phase and thus preventing potential gaps in monitoring coverage.

Despite these promising results, several limitations of the current study should be acknowledged. First, while specificity was assessed using seven key co-occurring species, the high biodiversity of the mangrove ecosystem necessitates future validation with a broader array of non-target organisms to definitively rule out cross-reactivity with rarer endemic species. Second, because our assays were primarily validated using high-quality genomic DNA extracted under controlled laboratory conditions, their performance—particularly the LAMP-LFD assay—warrants further evaluation using crude DNA extraction methods or direct tissue lysis. This is especially important given that the potential inhibitory effects of environmental contaminants in field samples remain fully unexplored. Finally, the field validation was conducted using a relatively limited sample size. Although these samples served as an initial proof-of-concept for field applicability, the small sample size and potentially narrow geographic coverage may not fully reflect natural variations in target density or the genetic diversity of *H. puera* populations across different regions. Addressing these constraints will be the focus of future work to optimize these assays for robust, large-scale field applications. Looking ahead, integrating these molecular tools with conventional monitoring techniques—such as light trapping and radar monitoring—offers a promising approach to better understand the drivers of *H. puera* outbreaks. Future efforts should focus on validating the LAMP-LFD assay for use with environmental DNA (eDNA) or bulk insect samples collected from traps. Overcoming current challenges related to sample preparation and field validation will be essential to advancing these assays from laboratory research to practical tools for the protection of mangrove ecosystems.

## 5. Conclusions

This study successfully developed two efficient molecular detection methods, SS-PCR and LAMP, targeting the mitochondrial *COI* gene for the rapid identification of the invasive pest *H. puera*. Both assays demonstrated exceptional specificity and stability, accurately distinguishing *H. puera* from non-target species across different developmental stages and geographical populations. While the SS-PCR assay provides a reliable solution for laboratory-based confirmation, the LAMP assay offers superior sensitivity (detection limit of 8.3 fg/µL) and efficiency. Furthermore, integrating LAMP with LFD technology enables convenient, instrument-free visual detection, making it highly suitable for field-based surveillance in resource-limited environments. This assay shows significant potential for integration into national quarantine programs as a rapid screening tool, substantially enhancing interception accuracy and efficiency to prevent the cross-border spread of this pest. Furthermore, this technology can be incorporated into digital monitoring systems, empowering frontline personnel to achieve early outbreak warning and precise forestry control, thereby enabling more effective field management. Collectively, these techniques overcome the limitations of morphological identification, providing powerful tools for the early detection, population monitoring, and timely control of *H. puera*, thereby contributing to the protection of mangrove and forest ecosystems.

## Figures and Tables

**Table 1:** Sampling Sites of *H. puera*.

Population	Sampling Site	Sampling Date	Latitude and Longitude	Elevation (m)
GXBH	Beihai, Guangxi	June 2024	21°29′58″ N, 109°45′38″ E	26
GDZJ	Zhanjiang, Guangdong	June 2024	21°29′42″ N, 109°8′1″ E	1
GDYJ	Yangjiang, Guangdong	June 2024	21°59′20″ N, 112°9′18″ E	13
YNBS	Baoshan, Yunnan	August 2024	24°20′47″ N, 99°1′58″ E	754

**Table 2:** *COI* gene primers used in this study.

Primers	Sequences (5′–3′)	Length (bp)	Production (bp)	Annealing (°C)
HPCOIF-1	TGAGCAGGAATAGTGGGGAC	20	603	59
HPCOIR-1	TCCCCCTGCAGGATCAAAAA	20		
HPCOIF-2	TGAGCAGGAATAGTGGGAACATCT	24	598	58
HPCOIR-2	GGATCTCCTCCTCCTGCAGG	20		
HPCOIF-3	AGGATTTGTTGTTTGAGCTCACCA	24	602	58
HPCOIR-3	ACGAATGTTCTGCAGGGGGA	20		

**Table 3:** Sequences of the LAMP primer set designed for the detection of *H. puera*.

Name	Sequence	Length (bp)	Production (bp)
F3	CAGGATCATTAATTGGAGATGA	22	233
B3	CCATTTTCAACAATTCTTCTTGA	23	
FIP	CAAGTCAATTTCCAAATCCTCCAATAATTGTTACAGCTCATGCA	44	
BIP	GAGCACCTGATATAGCTTTTCCAATAAAGTTAAAGATGGGGGTAA	45	

## Data Availability

The data presented in this study are available within the article and its Supplementary Materials.

## Supplementary Materials

The following supporting information can be downloaded at: https://www.mdpi.com/article/10.3390/biology15060473/s1. Figure S1. Sequence alignment of the target regions for *Hyblaea puera* and its closely related species, including the positions of LAMP and PCR primers. Figure S2. Original agarose gel electrophoresis images.

## Abbreviations

The following abbreviations are used in this manuscript:

PCGs	Protein-coding genes
SS-PCR	Species-Specific PCR
LAMP	Loop-mediated isothermal amplification
*COI*	Mitochondrial cytochrome c oxidase subunit I
*Pi*	Nucleotide diversity
