# Supplementary material for: Development and Application of Two Rapid Molecular Detection Assays for Hyblaea puera Cramer (Lepidoptera: Hyblaeoidea), a Major Pest of Mangroves and Teak
Source: Biology (Basel). 2026 Mar 15;15(6):473. doi: 10.3390/biology15060473 (PMC13023812; doi:10.3390/biology15060473)
Supplement: Supplementary file 1 [file biology-15-00473-s001.zip › biology-4132695-supplementary.pdf]

biology

Supplementary Materials

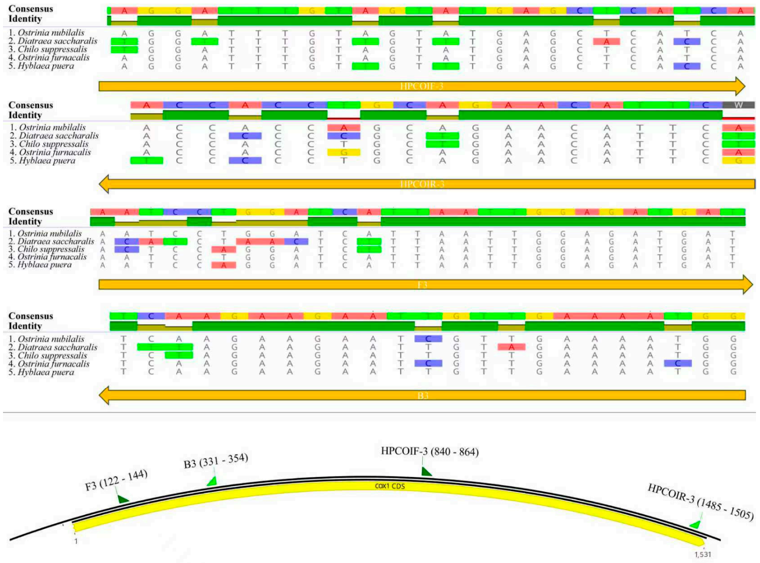

Figure S1. Sequence alignment of the target regions for *Hyblaea puera* and its closely related species, including the positions of LAMP and PCR primers.

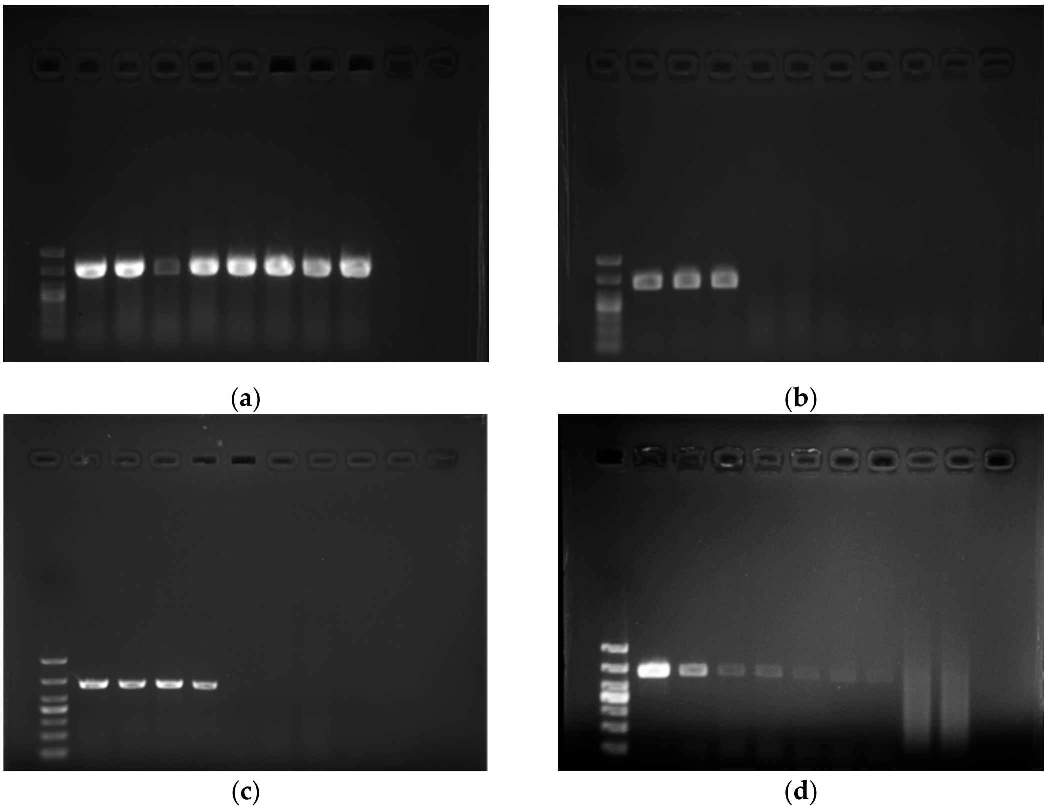

Figure S2. Original agarose gel electrophoresis images

Disclaimer/Publisher's Note: The statements, opinions and data contained in all publications are solely those of the individual author(s) and contributor(s) and not of MDPI and/or the editor(s). MDPI and/or the editor(s) disclaim responsibility for any injury to people or property resulting from any ideas, methods, instructions or products referred to in the content.

https://doi.org/10.3390/biology15060473
